# Effects of MicroRNA-195-5p on Biological Behaviors and Radiosensitivity of Lung Adenocarcinoma Cells via Targeting HOXA10

**DOI:** 10.1155/2021/4522210

**Published:** 2021-12-07

**Authors:** Cheng Yuan, Rui Bai, Yanping Gao, Xueping Jiang, Shuying Li, Wenjie Sun, Yangyi Li, Zhengrong Huang, Yan Gong, Conghua Xie

**Affiliations:** ^1^Department of Radiation and Medical Oncology, Zhongnan Hospital of Wuhan University, Wuhan, Hubei 430071, China; ^2^Department of Gynecological Oncology, Zhongnan Hospital of Wuhan University, Wuhan 430071, China; ^3^Department of Biological Repositories, Zhongnan Hospital of Wuhan University, Wuhan, Hubei 430071, China; ^4^Hubei Key Laboratory of Tumour Biological Behaviors, Zhongnan Hospital of Wuhan University, Wuhan, Hubei 430071, China; ^5^Hubei Cancer Clinical Study Center, Zhongnan Hospital of Wuhan University, Wuhan, Hubei 430071, China

## Abstract

**Objective:**

To explore the effects of miR-195-5p and its target gene HOXA10 on the biological behaviors and radiosensitivity of lung adenocarcinoma (LUAD) cells.

**Methods:**

The effects of miR-195-5p on LUAD cell proliferation, migration, invasion, cycle arrest, apoptosis, and radiosensitivity were investigated by *in vitro* experiments. The bioinformatics analysis was used to assess its clinical value and predict target genes. Double-luciferase experiments were used to verify whether the miR-195-5p directly targeted HOXA10. A xenograft tumor-bearing mouse model was used to examine its effects on the radiosensitivity of LUAD *in vivo*.

**Results:**

Both gain- and loss-of-function assays demonstrated that miR-195-5p inhibited LUAD cell proliferation, invasion, and migration, induced G1 phase arrest and apoptosis, and enhanced radiosensitivity. Double-luciferase experiments confirmed that miR-195-5p directly targeted HOXA10. Downregulation of HOXA10 also inhibited LUAD cell proliferation, migration, and invasion, induced G1 phase arrest and apoptosis, and enhanced radiosensitivity. The protein levels of *β*-catenin, c-myc, and Wnt1 were decreased by miR-195-5p and increased by its inhibitor. Moreover, the effects of the miR-195-5p inhibitor could be eliminated by HOXA10-siRNA. Furthermore, miR-195-5p improved radiosensitivity of LUAD cells *in vivo*.

**Conclusion:**

miR-195-5p has excellent antitumor effects via inhibiting cancer cell growth, invasion, and migration, arresting the cell cycle, promoting apoptosis, and sensitizing LUAD cells to X-ray irradiation by targeting HOXA10. Thus, miR-195-5p may serve as a potential candidate for the treatment of LUAD.

## 1. Introduction

Lung cancer is the leading cause of cancer-related deaths worldwide, with over 2 million new cases diagnosed [1]. As the most common pathological type, lung adenocarcinoma (LUAD) has strong invasive ability and generally poor prognosis [2, 3]. As a primary epithelial tumor of the lung, LUAD pathogenesis involves many factors such as environment, heredity, and living habits [4]. However, its cause has not yet been fully elucidated. Because LUAD often manifests as peripheral lung cancer clinically, the early clinical symptoms are often atypical, leading to the difficulty of early diagnosis. Some LUAD have metastasized or invaded to the pleura at the time of diagnosis, resulting in a generally poor prognosis for patients [5, 6]. Therefore, it is very important and urgent to identify effective biomarkers for its early diagnosis.

Radiotherapy is a local treatment of a tumor, which is widely used in the clinical treatment of LUAD. Nevertheless, many patients have radiotherapy resistance, which often limits the therapeutic effects of radiotherapy. Previous researches reported that radiotherapy caused changes in the expression profile of miRNAs in LUAD patients, and these miRNAs might be related to their radiosensitivity [7–9].

Previous studies [10–12] have confirmed that miR-195-5p had significant antitumor effects. Feng et al. [10] found that miR-195-5p increased chemosensitivity and apoptosis in chemoresistant colorectal cancer cells. A similar phenomenon was also observed [11], which indicated that miR-195-5p could reduce chemoresistance by inhibiting the stem cell-like ability of colorectal cancer cells. Chai et al. found that miR-195-5p, acting as tumor suppressors of melanoma, inhibited A375 cell proliferation, migration, and invasion, arrested the cell cycle, and induced cell apoptosis [12]. In our previous work, we revealed that miR-195-5p had high diagnostic values for LUAD [13]. Higher expression of miR-195-5p indicated better LUAD prognosis. Therefore, in our current research, we explored its roles in response to radiation in LUAD cells and mouse model.

## 2. Materials and Methods

### 2.1. Target Prediction and Function of miR-195-5p

Three different databases (TargetScan, miRDB, and DIANA-TarBase) were adopted to predict miRNA target genes. Based on TCGA-LUAD expression profile data, the upregulated genes in LUAD tissues (log_2_FC > 1, adjust *P* < 0.05) were selected. The ROC diagnostic test and Kaplan-Meier plots were then used to evaluate the diagnostic and prognostic values. The cut-off value was AUC > 0.8, HR > 1, and log rank *P* < 0.05. In order to further explore the function of target genes, single gene set enrichment analysis (GSEA) was used to explore the functional signaling pathways related to the target genes.

### 2.2. Cells Culture and Transfection

PC9 and A549 cells were cultured in RPMI medium (Gibco, USA) containing 10% fetal calf serum. The miR-195-5p mimic, inhibitor, and negative control (NC) were provided by RiboBio, China, and the corresponding transfection reagent was Lipofectamine 2000 (Invitrogen, USA). Three siRNA duplexes (GenePharma, China) were designed to target human HOXA10, transfected with jetPRIME (PolyPlus-transfection).

### 2.3. Total RNA Extraction and qRT-PCR

TRIzol was used to extract total RNA directly from PC9 and A549 cells. The PrimeScript™ RT Reagent Kit with gDNAEraser was adopted to detect the levels of mature miR-195-5p. The levels of mRNAs were detected by PrimeScript™ RT Reagent Kit and gDNAEraser and SYBR Advantage qPCR Premix (Takara). GADPH and U6 were used as references.  Table 1 provides all PCR-related primer sequences.

### 2.4. Cell Proliferation, Migration, and Invasion Assays

The cell viability at different time points was measured by the Cell Counting Kit-8 (CCK-8, Beyotime). The migration of LUAD cells was examined by the wound healing assay. LUAD cells were cultured and incubated until the cell confluence was over 90% in 6-well plates. A clean pipette tip (200 *μ*L) was applied to create a scratch in the middle of the cell monolayer. After being cultured with fresh serum-free medium for 48 h, the wound was photographed with an inverted microscope. DiI (5 *μ*M, Google Bio, China) was used 30 min before the observation. For invasion assays, the lower chambers of transwell were filled with 700 *μ*L complete medium. The 1 × 10^5^ LUAD cells were seeded into the upper chambers. The invasive cells were stained with 1% crystal violet solution. Images of invading cells were captured under an inverted microscope.

### 2.5. Cell Cycle and Apoptosis Assays

After transfection or irradiation (4 Gy) for 48 h, the apoptotic/necrotic cells (1 × 10^3^ cells/*μ*L) were suspended in 400 mL binding buffer and stained using 5 *μ*L Annexin V/FITC for 15 min and 10 *μ*L PI for 5 min by using Annexin V/PI staining kit (BestBio, China). For cell cycle assays, the cells (2 × 10^5^ cells/well) were stained with 1 mL DNA staining solution (containing RNase A: 0.1 mg/mL) and 10 *μ*L PI (0.5 mg/mL) mixed solution by using a cell cycle staining kit (MultiSciences Biotech, China). Cell cycle and apoptosis assays were measured by using BD FACSVerse™.

### 2.6. Colony Survival Assay

PC9 and A549 cells, transfected with mimics, inhibitors, siRNA, and corresponding NC, were seeded in 6-well plates (100, 200, 400, 600, 800, 1000, and 2000 cells/well) and exposed to the following radiation (0, 1, 2, 4, 6, 8, and 10 Gy) correspondingly. After 13 days, the colonies were counted and cell survival was evaluated. Specifically, colonies with more than 50 cells were counted, and the survival fraction was calculated by the ratio of the colony number and seed cell number.

### 2.7. Luciferase Assay

To construct a HOXA10 3′-untranslated region- (UTR-) luciferase plasmid, the HOXA10 3′-UTR fragments containing the miR-195-5p-binding (HOXA10-3′UTR-wt) or HOXA10 3′-UTR-mutated (HOXA10-3′UTR-mut) site were inserted into the pSI-Check2 vector, which contains a luciferase reporter gene. After constructing the HOXA10 3′-UTR-luciferase plasmid, 293T cells were transfected. Then, Firefly and Renilla luciferase activities were tested.

### 2.8. Immunoblotting

The antibodies of BCL2, Bax, MMP-2, MMP-9, cyclin D1, and c-myc were from Wuhan Sanying, China. The antibodies of *β*-catenin and Wnt1 were purchased from Cell Signaling Technology, USA, and the antibody of HOXA10 was from Abcam, UK. The proteins were extracted by using RIPA buffer with 1% PMSF and then loaded onto an SDS-PAGE minigel. After being transferred onto PVDF membranes, the primary and corresponding secondary antibodies were used to label the target proteins. GAPDH was used as an endogenous protein for normalization.

### 2.9. Tumor Formation in Nude Mice

The nude mice (BALB/c, 4-6 weeks old) were provided by Vital River Laboratory Animal Technology, China. All mouse feeding and operation processes followed the Experimental Animal Welfare Ethics Committee of Zhongnan Hospital of Wuhan University. PC9 was used to construct a subcutaneous implant tumor model (6 × 10^6^ cells/mouse). miR-195-5p agomir and miR agomir NC were provided by RiboBio, China. Fifteen days after cell injection, they were administered by intratumoral injection (2 nmol/30 *μ*L PBS) for 5 consecutive days when the tumor volume was approximately 100 mm^3^. The mice were divided into 2 groups. One group had no additional treatment, and the other group received X-ray irradiation (10 Gy once). Nude mice were executed via anesthesia at 30 days after irradiation, and the tumors were removed for photographing and the tumor volumes were calculated.

### 2.10. Statistical Analysis

The results were presented as means ± standard deviation (SD). One-way analysis of variance (ANOVA) or Student's *t*-test was performed to estimate the significance between groups. *P* < 0.05 was considered statistically significant.

## 3. Results

### 3.1. miR-195-5p Inhibited Proliferation, Invasion, and Migration of LUAD Cells

**Figure 1:**
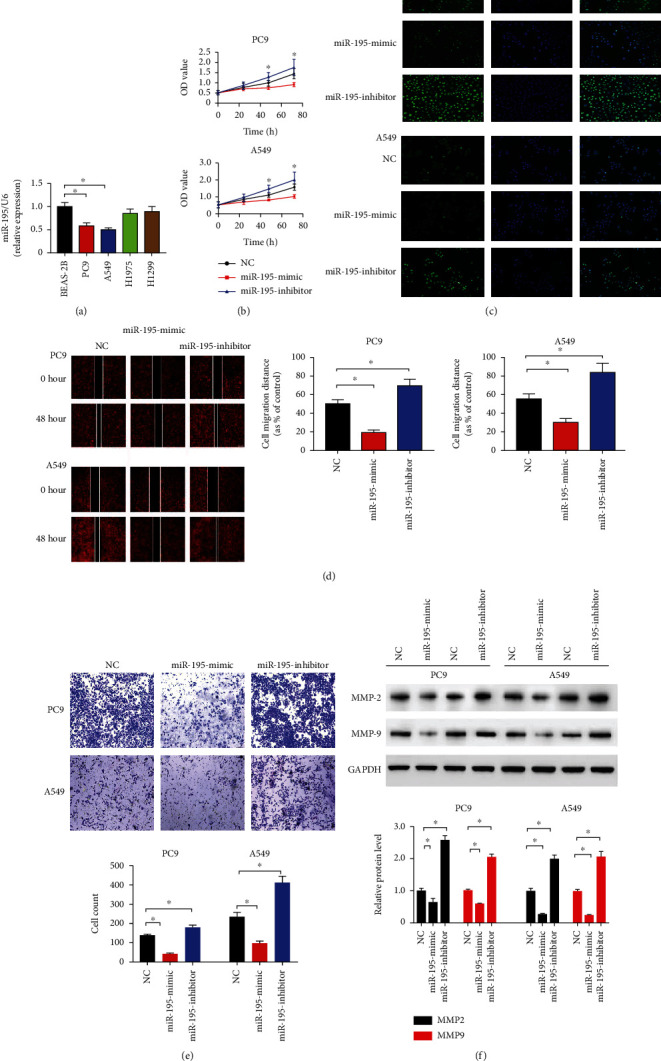
miR-195-5p inhibited proliferation, invasion, and metastasis of LUAD cells. (a) The expression levels of miR-195-5p in BASE-2B, PC9, A549, H1975, and H1299 cell lines. (b) CCK-8 assays of PC9 and A549 cells transfected with the miR-195-5p mimic, inhibitor, and NC. (c) Representative immunofluorescent images of Ki67. (d) Representative images and quantification of wound healing assays in PC9 and A549 cells. (e) Representative images and quantification of transwell migration assays of PC9 and A549 cells. (f) Representative immunoblotting of MMP2 and MMP9 in A549 and PC9 cells. *n* = 3; ^∗^*P* < 0.05 vs. BASE-2B or NC.

Compared with the normal lung epithelial cell line (BASE-2B), miR-195-5p levels were significantly lower in A549 and PC9 cells (Figure 1(a)). The results of cell viability assays suggested that the proliferation of A549 and PC9 cells was restrained by the miR-195-5p mimic (Figure 1(b)), which was in accordance with the results of immunofluorescence of Ki67 (Figure 1(c)). The inhibitors of miR-195-5p induced LUAD cell proliferation and increased Ki67 staining. The results of migration and invasion assays indicated that the miR-195-5p mimic reduced PC9 and A549 cell migration, while its inhibitors had the opposite effects (Figures 1(d) and 1(e)). Immunoblotting confirmed that the miR-195-5p mimic downregulated the protein levels of MMP2 and MMP9, while its inhibitors induced these 2 proteins in A549 and PC9 cells (Figure 1(f)).

### 3.2. miR-195-5p Enhanced Radiosensitivity

**Figure 2:**
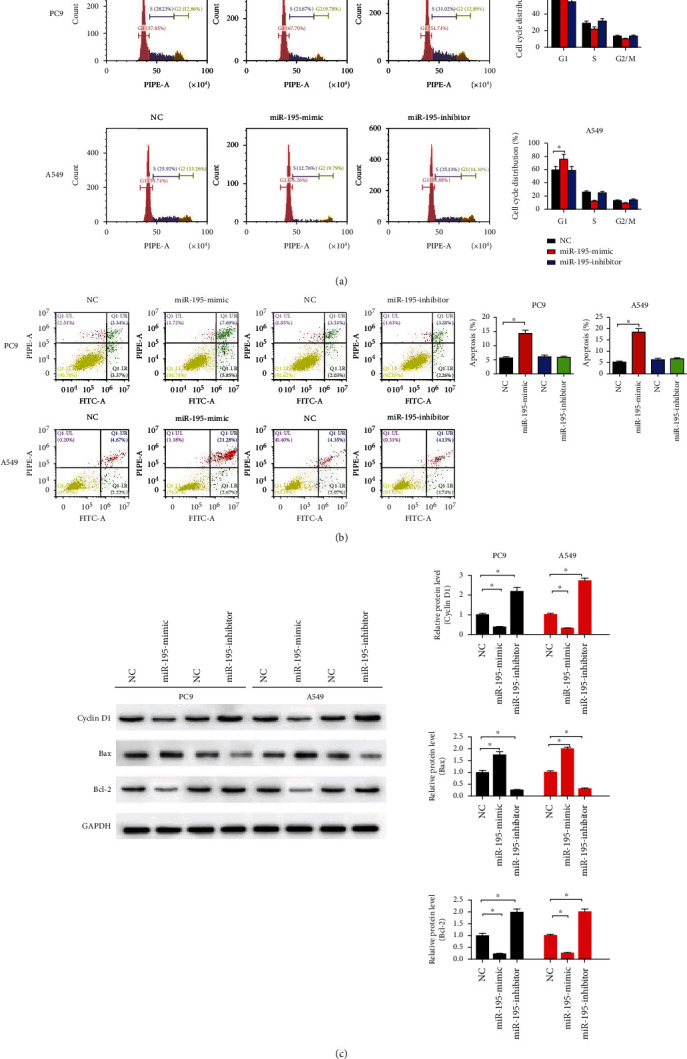
miR-195-5p induced apoptosis and blocked the cell cycle. (a) Flow cytometry shows the percentage of cells at different cell cycle phases. (b) Cell apoptosis was detected by flow cytometry at 48 h after being transfected with the miR-195-5p mimic, inhibitor, and NC. (c) The expression levels of cycle- and apoptosis-related proteins (cyclin D1, Bax, and Bcl-2) were measured by immunoblotting. *n* = 3; ^∗^*P* < 0.05 vs. NC.

The miR-195-5p mimic increased the numbers of PC9 and A549 cells at the G1 phase, suggesting that the miR-195-5p mimic arrested cells at the G1 phase (Figure 2(a)). In addition, the miR-195-5p mimic also significantly induced LUAD cell apoptosis (*P* < 0.05, Figure 2(b)). The results of immunoblotting demonstrated that miR-195-5p overexpression increased the levels of Bax and reduced cyclin D1 and Bcl-2 expression in PC9 and A549 cells (Figure 2(c)).

**Figure 3:**
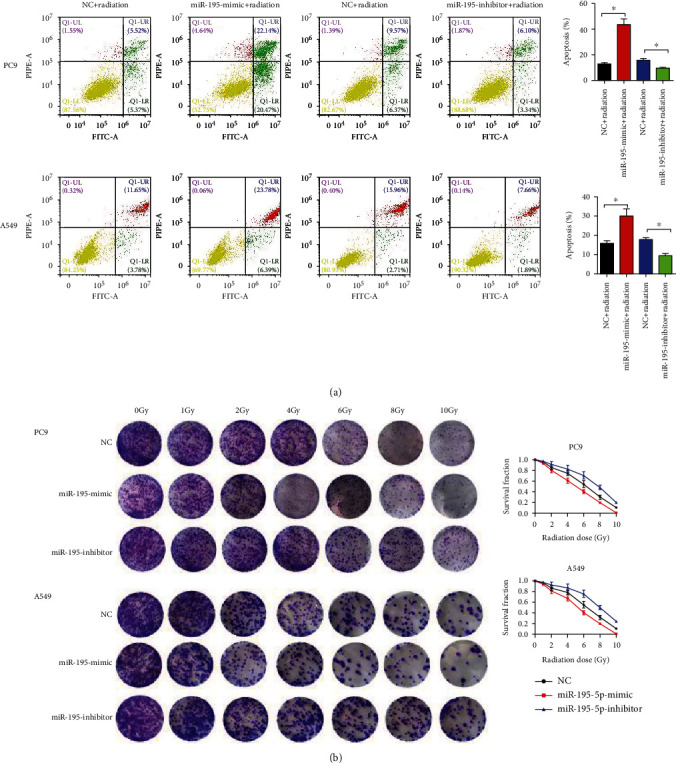
miR-195-5p enhanced radiosensitivity of LUAD cells. (a) Cell apoptosis was detected by flow cytometry at 48 h after radiation (4 Gy). (b) Survival fractions were calculated after treatment with 0, 2, 4, 6, 8, and 10 Gy of ionizing radiation. *n* = 3, ^∗^*P* < 0.05 vs. NC.

To examine whether miR-195-5p affected radiosensitivity of LUAD cells, PC9 and A549 cells were exposed to ionizing radiation (4 Gy). The apoptosis levels of PC9 and A549 cells were measured, and miR-195-5p enhanced LUAD cell apoptosis induced by X-ray (Figure 3(a)). The colony survival assay showed that the miR-195-5p mimic reduced the survival fractions of PC9 and A549 cells at each dose (Figure 3(b)).

### 3.3. miR-195-5p Targeted HOXA10

Based on TCGA-LUAD profile data, the differentially expressed microRNAs (DEMs) and differentially expressed genes (DEGs) in LUAD tissues were selected. Three different databases (TargetScan, miRDB, and DIANA-TarBase) were adopted to predict miRNA target genes. When DEMs were matched to GEGs, 34 target genes were screened out (Figure S1). Among them, 16 target genes were the target genes of miR-195-5p. Except TMEM00, RS1, and OSCAR, the remaining genes (CEP55, PSAT1, CHEK1, KIF23, CCNE1, CLSPN, CDC25A, E2F7, CBX2, HOXA10, SALL1, TGFBR3, and RET) were upregulated in LUAD tissues (Figure S2). The clinical values of these upregulated target genes were evaluated by diagnostic efficacy and prognostic analysis (Figure S3). The results suggested that HOXA10 might be a hub target gene, which had an important impact on the prognosis of LUAD patients.

**Figure 4:**
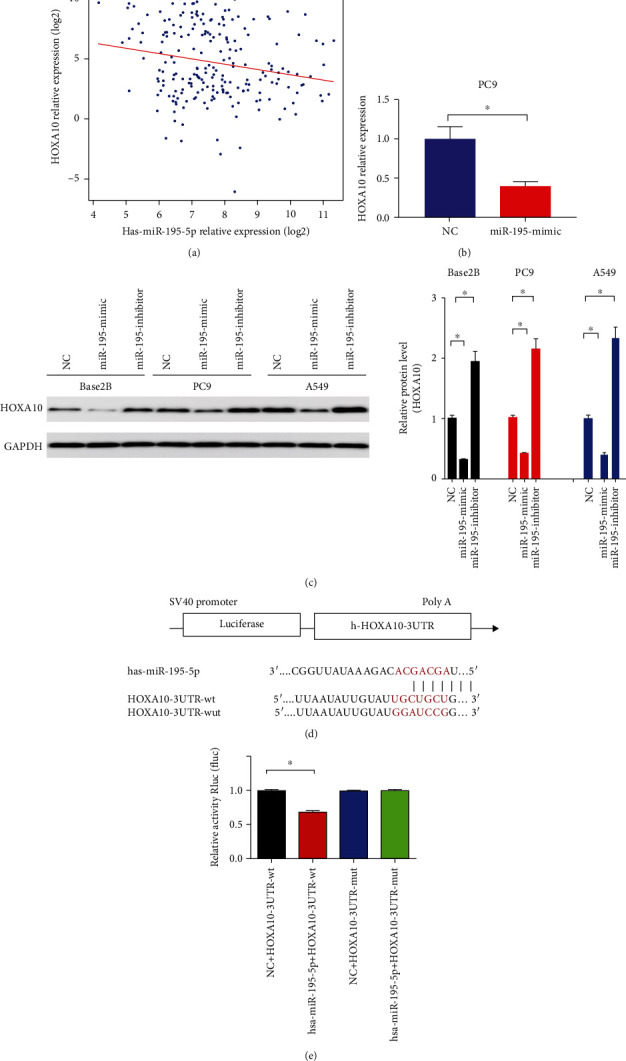
miR-195-5p targeted HOXA10. (a) The expression levels of miR-195-5p and HOXA10 showed a negative correlation (*r* = −0.2, *P* < 0.05) in TCGA database. The relative mRNA (b) and protein (c) levels of HOXA10 were detected in PC9 and A549 cells transfected with the miR-195-5p mimic, inhibitor, and NC. (d, e) Dual-luciferase reporter experiments revealed that miR-195-5p directly bound to HOXA10 and reduced its expression. *n* = 3; ^∗^*P* < 0.05 vs. NC.

Pearson's correlation analysis with TCGA database revealed that the levels of HOXA10 were suppressed by miR-195-5p in LUAD (*r* = −0.2, Figure 4(a)). In LUAD cells, both mRNA and protein levels of HOXA10 were downregulated by the miR-195-5p mimic (Figures 4(b) and 4(c)). Dual-luciferase reporter experiments confirmed that the miR-195-5p mimic downregulated the luciferase expression of HOXA10-3'UTR-wt (*P* < 0.001), indicating a direct targeting. Moreover, the miR-195-5p mimic failed to downregulate the luciferase level of HOXA10-3′UTR-mut (Figures 4(d) and 4(e)), suggesting that miR-195-5p directly targeted HOXA10.

### 3.4. Effects of HOXA10 on Proliferation, Migration, and Invasion of LUAD Cells

**Figure 5:**
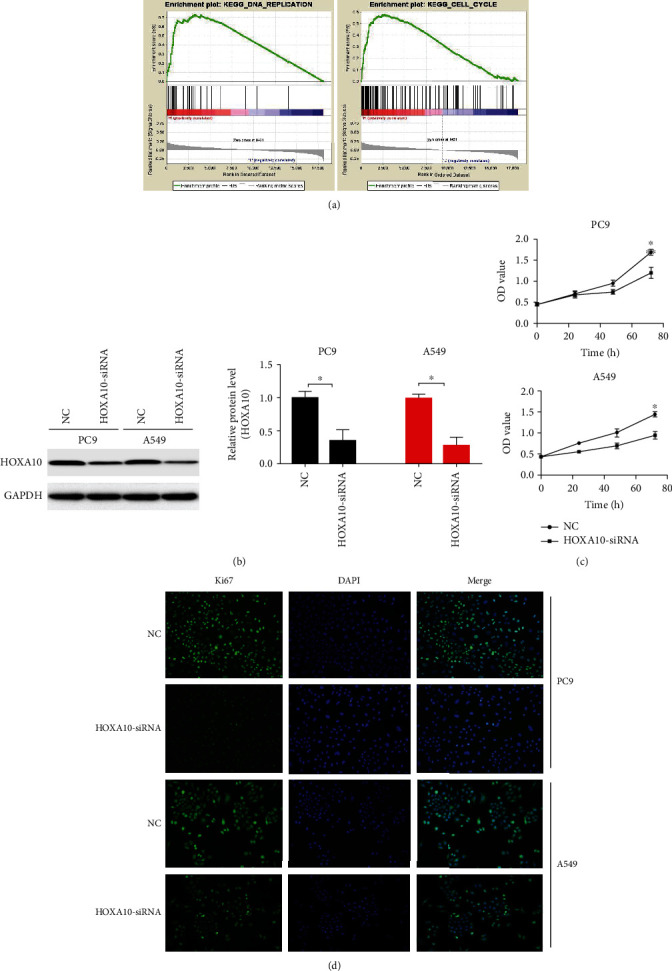
HOXA10 downregulation inhibited LUAD cell proliferation. (a) The results of GSEA predicted that DNA replication and cell cycle-related signal pathways were activated in the HOXA10 high-expression group. (b) The protein levels of HOXA10 were detected in PC9 and A549 cells transfected with HOXA10-siRNA and NC. (c) CCK-8 assays of PC9 and A549 cells transfected with HOXA10-siRNA and NC. (d) Representative immunofluorescent images of Ki67 in PC9 and A549 cells transfected with HOXA10-siRNA and NC. *n* = 3; ^∗^*P* < 0.05 vs. NC.

To explore the biological functions of the HOXA10, single gene set enrichment analysis was performed. The logarithm for the fold difference of 18,148 protein coding genes between the high- and low-expression groups was analyzed. The results showed that DNA replication and cell cycle-related signal pathways were activated in the HOXA10 high-expression group (Figure 5(a)). The above results further suggested the importance of HOXA10 in the occurrence and development of LUAD.

**Figure 6:**
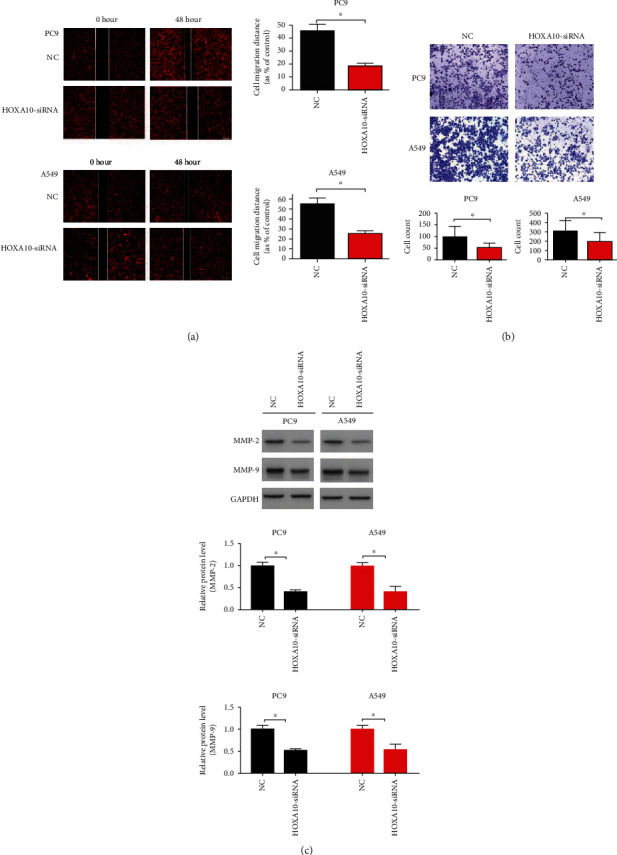
HOXA10 downregulation inhibited invasion and metastasis of LUAD cells. (a) Representative images of the wound healing assay in PC9 and A549 cells transfected with HOXA10-siRNA and NC. (b) Representative images of the modified Boyden chamber assay in PC9 and A549 cells transfected with HOXA10-siRNA and NC. (c) Immunoblotting results demonstrated that MMP2 and MMP9 protein levels were decreased in the HOXA10-siRNA group. *n* = 3; ^∗^*P* < 0.05 vs. NC.

After the expression of HOXA10 was downregulated with siRNAs (Figure 5(b)), the proliferation of PC9 and A549 cells was inhibited (Figure 5(c)). This phenomenon can be confirmed by the detection of Ki67 immunofluorescence (Figure 5(d)). HOXA10-siRNA inhibited LUAD cell migration (Figure 6(a)) and invasion (Figure 6(b)). Immunoblotting results showed that MMP2 and MMP9 protein levels were decreased in the HOXA10-siRNA group compared with NC (Figure 6(c)).

### 3.5. HOXA10 Decreased Radiosensitivity of LUAD Cells

**Figure 7:**
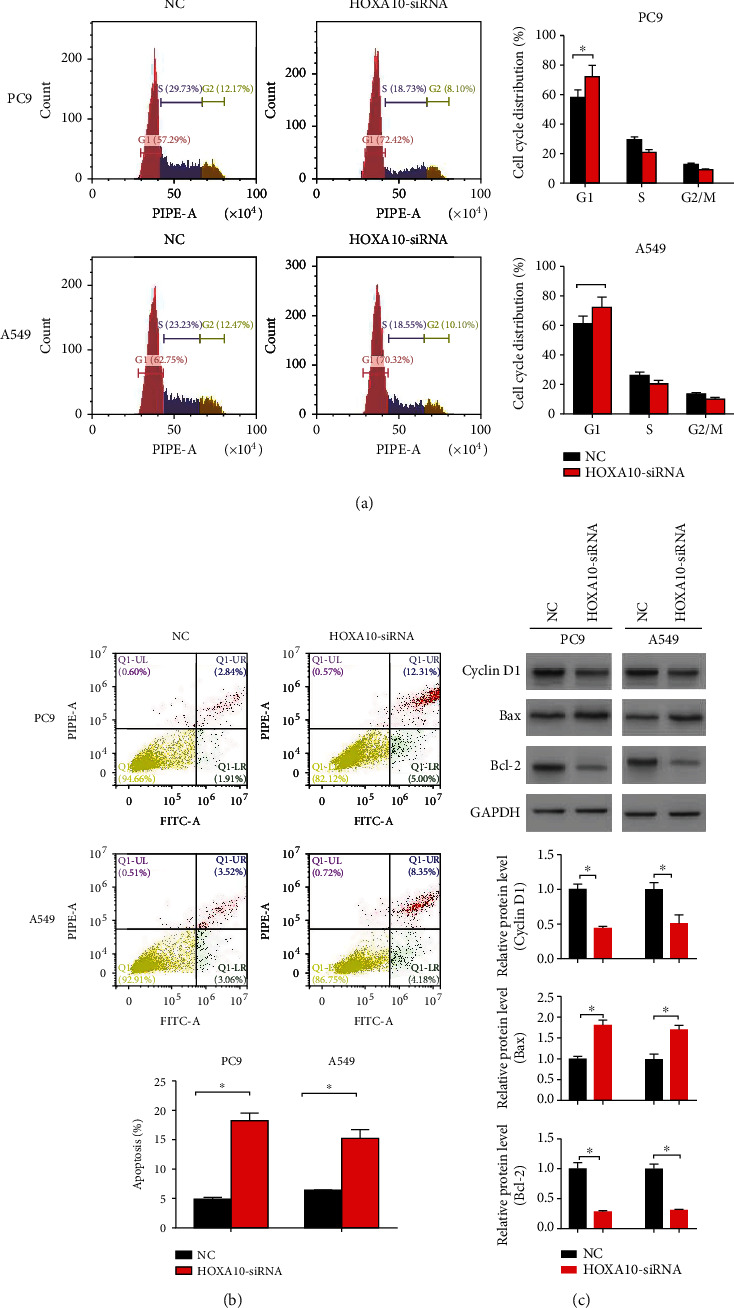
HOXA10 downregulation induced LUAD cell apoptosis and blocked the cell cycle. (a) The cell cycle was detected by flow cytometry. (b) Apoptosis was detected by flow cytometry. (c) The levels of cycle- and apoptosis-related proteins (cyclin D1, Bax, and Bcl-2) were measured by immunoblotting. *n* = 3; ^∗^*P* < 0.05 vs. NC.

The results of cell cycle analysis indicated that HOXA10 knockdown blocked the cell cycle at the G1 phase, and the rate of apoptosis was significantly increased (Figures 7(a) and 7(b)). Immunoblotting results showed a decrease in cyclin D1 and Bcl-2 and an increase in Bax protein levels in the HOXA10-siRNA group (Figure 7(c)).

**Figure 8:**
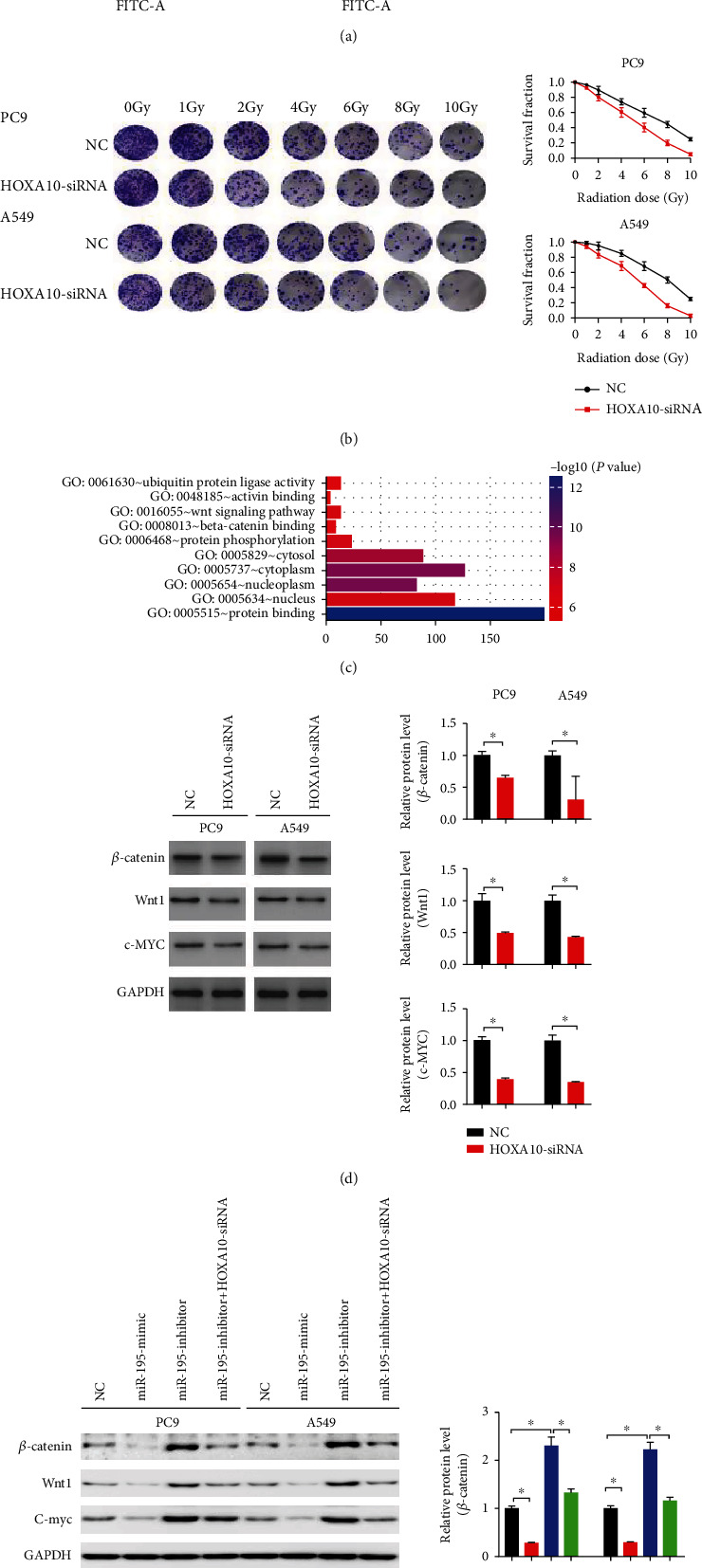
HOXA10 downregulation enhanced radiosensitivity of LUAD cells. (a) Cell apoptosis was detected by flow cytometry at 48 h after radiation (4 Gy). (b) Colony formation assays and survival fractions were calculated after treatment with 0, 2, 4, 6, 8, and 10 Gy of ionizing radiation. (c) The enrichment analysis indicated that HOXA10 might be involved in the regulation of the Wnt pathway. (d) Representative immunoblots of *β*-catenin, c-myc, and Wnt1 in PC9 and A549 cells transfected with HOXA10-siRNA and NC. (e) Representative immunoblots of *β*-catenin, c-myc, and Wnt1 in PC9 and A549 cells transfected with the miR-195-5p mimic, inhibitor, inhibitor+OXA10-siRNA, and NC. *n* = 3; ^∗^*P* < 0.05 vs. NC.

Moreover, HOXA10 knockdown increased the rate of apoptosis induced by radiation (Figure 8(a)). Colony formation assays confirmed that HOXA10 deficiency improved PC9 and A549 cell radiosensitivity (Figure 8(b)). It was worth noting that the enrichment analysis results suggested that HOXA10 might be involved in the regulation of the Wnt pathway (Figure 8(c)). To confirm this prediction, we examined the expression of *β*-catenin, c-myc, and Wnt1, and their protein levels were inhibited by HOXA10-siRNA (Figure 8(d)).

Therefore, HOXA10 may regulate the tumor biological behaviors and radiosensitivity of the LUAD cells through the Wnt/*β*-catenin pathway. To further clarify whether miR-195-5p regulated the Wnt/*β*-catenin pathway through HOXA10, we investigated its effects on the expression of Wnt1 and *β*-catenin in LUAD cells. It was found that the miR-195-5p mimic inhibited the Wnt/*β*-catenin pathway, while its inhibitors activated this pathway. Moreover, the effects of miR-195-5p inhibitors on the Wnt/*β*-catenin pathway could be eliminated by HOXA10-siRNA. Therefore, miR-195-5p regulated the Wnt pathway through HOXA10 (Figure 8(e)).

### 3.6. miR-195-5p Enhanced the Radiosensitivity of Xenograft-Formed LUAD

**Figure 9:**
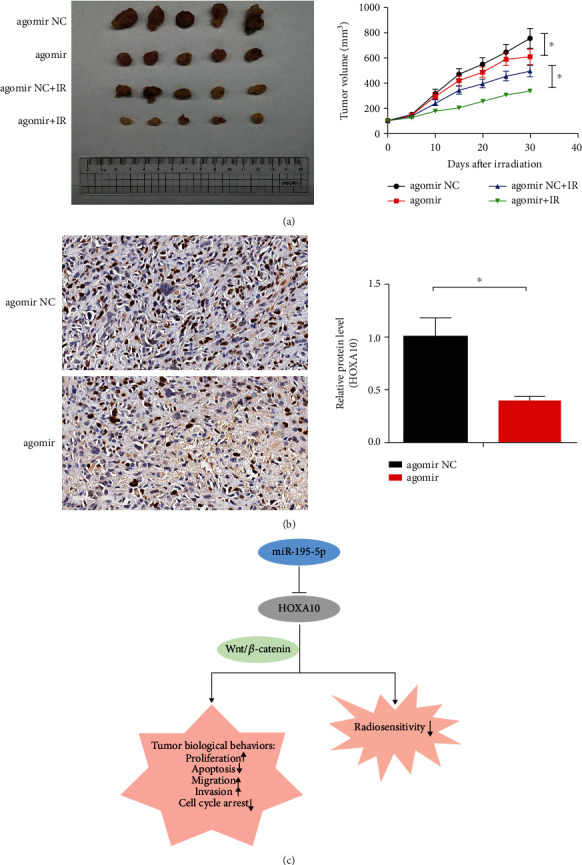
Effects of miR-195-5p on the radiosensitivity of the xenograft LUAD tumor. (a) Tumor volume in the nude mice. (b) Representative immunohistochemistry of HOXA10 in the LUAD tissues from nude mice. (c) Hypothesis diagram showing that miR-195-5p targeted HOXA10 to hinder the corresponding cytological behaviors of LUAD cells and enhance radiosensitivity. *n* = 5; ^∗^*P* < 0.05 vs. NC.

The tumor volume in the mice injected with miR-195-5p agomir combined with irradiation was lower than that of agomir NC and radiation alone (Figure 9(a)). In addition, the expression levels of HOXA10 in the tumor tissues of nude mice treated with miR-195-5p agomir were lower than those in the agomir NC tumors (Figure 9(b)).

## 4. Discussion

Radiotherapy is widely used in the clinical treatment of LUAD. However, some patients have radiotherapy resistance at the beginning or during treatment, which is a challenge. Researchers try to explain the mechanism of radioresistance, including autophagy [14], tumor stem cells [15], and abnormal activation of signal pathways [16]. Previous studies have reported that miRNAs affect radiation damage to cells in different tumors, indicating that miRNAs may play important roles in tumor radiosensitivity [17–19].

Our current study supported that increased miR-195-5p in LUAD cells considerably decreased cell growth, migration, and invasion. Subsequent studies found that miR-195-5p could also increase the cell proportion of the G1 phase in the cell cycle, induce apoptosis, and improve radiosensitivity. Moreover, miR-195-5p could directly regulate HOXA10 expression via targeting its 3′-UTR. It downregulated HOXA10, *β*-catenin, c-myc, and Wnt1 in PC9 and A549 cells. Finally, miR-195-5p suppressed LUAD growth and enhanced radiosensitivity *in vivo*.

More and more researches supported that miRNAs could be used as biomarkers for malignant tumors. miRNAs were widely present in body fluids and tissues, and their abnormal expression or distribution has important impacts on the biological behaviors [20, 21]. Previous studies suggested that miR-195-5p was proposed to be a biomarker of lung cancer [22]. Our bioinformatics results also showed that miR-195-5p might be a tumor suppressor gene in lung cancer [13], and our *in vitro* experiments were consistent with previous results in non-small-cell lung cancer [23]. Guan et al. reported that HOXA10 mediated EMT in head and neck squamous cell carcinoma and was targeted by miR-195-5p [24]. However, their effects on radiosensitivity were to be investigated.

Previous researches reported that miRNAs affected cell apoptosis and regulated tumor cell radiosensitivity. For example, miR-148b promoted the cell apoptosis and enhances radiosensitivity [25], and miR-185 inhibited the apoptosis of gastric cancer cells [26]. Our studies found that overexpression of miR-195-5p enhanced LUAD radiosensitivity, evidenced by reduced survival fractions and induced apoptosis after miR-195-5p overexpression. In addition, the increase in the cell apoptosis rate was accompanied by induced expression of the proapoptotic molecule Bax, while the levels of Bcl-2 were downregulated. Therefore, it can be considered that miR-195-5p in LUAD cells might modify the outcome of radiation by the induction of apoptosis.

Dual-luciferase reporter experiments suggested that miR-195-5p directly targeted HOXA10. HOXA10 plays a vital function in embryonic development and cell proliferation and differentiation. Its expression levels are elevated in various tumors. For example, Plowright et al. found that HOXA10 was upregulated in NSCLC [27]. The expression levels of HOXA10 were negatively correlated with the invasion ability of gastric tumor cells. Moreover, Chu et al. reported that HOXA10 induced P53 to exhibit tumor suppressor genes [28]. Therefore, HOXA10 regulated tumor growth in a cancer-specific manner.

The specific functions of HOXA10 in LUAD need to be further explored. The CCK-8 and Ki67 immunofluorescence suggested that HOXA10 downregulation inhibited LUAD cell proliferation, migration, and invasion. The colony formation assay indicated that as radiation dose increased, the colony numbers gradually decreased. At the same dose level, HOXA10 knockdown significantly suppressed colony formation. These results indicated that HOXA10 deficiency induced LUAD cell apoptosis after radiation and reduced cell proliferation and survival. Our findings suggested that miR-195-5p enhanced the radiosensitivity of LUAD cells via inhibiting the HOXA10 expression.

The Wnt/*β*-catenin signaling pathway is closely related to the biological behaviors of the tumor. Yang et al. found that miR-183 inhibited osteosarcoma cell growth, migration, and invasion via regulating the Wnt/*β*-catenin signaling pathway [29]. Other studies [30–32] also suggested that this pathway was involved in the regulation of radiosensitivity. Interestingly, our results indicated that HOXA10 downregulation significantly inhibited the Wnt/*β*-catenin signaling pathway, as well as the biological behaviors of LUAD cells. Moreover, HOXA10 deficiency reversed Wnt/*β*-catenin signaling activation induced by the miR-195-5p inhibitor. Therefore, miR-195-5p hindered the activation of the Wnt/*β*-catenin signaling pathway via targeting HOXA10, thereby inhibiting the corresponding cytological behaviors of LUAD cells and enhancing radiosensitivity (Figure 9(c)).

Our studies had certain limitations. The results of our researches are to be confirmed with clinical evidence. Furthermore, the direct targeting of HOXA10 and how it affects the Wnt/*β*-catenin pathway need further exploration.

In summary, our results demonstrated that miR-195-5p inhibited biological behaviors and sensitized LUAD cells to X-ray irradiation via targeting HOXA10. It provided a novel idea to improve the treatment of LUAD, especially the efficacy of radiotherapy.

## Figures and Tables

**Table 1:** PCR primer sequence.

Primer name	Sequence (5′ to 3′)
miR-195-5p forward	GTCTAGCAGCACAGAAATA
miR-195-5p reverse	GTGCAGGGTCCGAGGT
miR-195-5p RT	GTCGTATCCAGTGCAGGGTCCGAGGTATTCGCACTGGATACGACGCCAA
U6 forward primer	CTCGCTTCGGCAGCACA
U6 reverse primer	AACGCTTCACGAATTTGCGT
HOXA10 forward	GGGTAAGCGGAATAAACT
HOXA10 reverse	GCACAGCAGCAATACAATA
GAPDH forward	GGAGCGAGATCCCTCCAAAAT
GAPDH reverse	GGCTGTTGTCATACTTCTCATGG

## Data Availability

The data used to support the findings of this study are available from the corresponding author upon request.

## Supplementary Materials

Supplementary 1 Supplemental Figure S1: Venn diagrams of differentially expressed microRNA targets. When matched to differentially expressed genes, 34 target genes were screened out.

Supplementary 2 Supplemental Figure S2: expression of the target genes was plotted for LUAD tumor and normal tissues (TCGA dataset). Except TMEM00, RS1, and OSCAR, the remaining genes (CEP55, PSAT1, CHEK1, KIF23, CCNE1, CLSPN, CDC25A, E2F7, CBX2, HOXA10, SALL1, TGFBR3, and RET) were upregulated in LUAD tissues.

Supplementary 3 Supplemental Figure S3: the target genes were evaluated by diagnostic efficacy and prognostic analysis. ROC curve analysis was performed based on TCGA dataset. Kaplan-Meier survival curves of the overall survival were shown in the Kaplan-Meier plotter (https://kmplot.com/analysis/).
