# Supplementary figures and images for: Effects of MicroRNA-195-5p on Biological Behaviors and Radiosensitivity of Lung Adenocarcinoma Cells via Targeting HOXA10
Source: Oxid Med Cell Longev. 2021 Dec 7;2021:4522210. doi: 10.1155/2021/4522210 (PMC8672108; doi:10.1155/2021/4522210)

Supplemental Figure S1

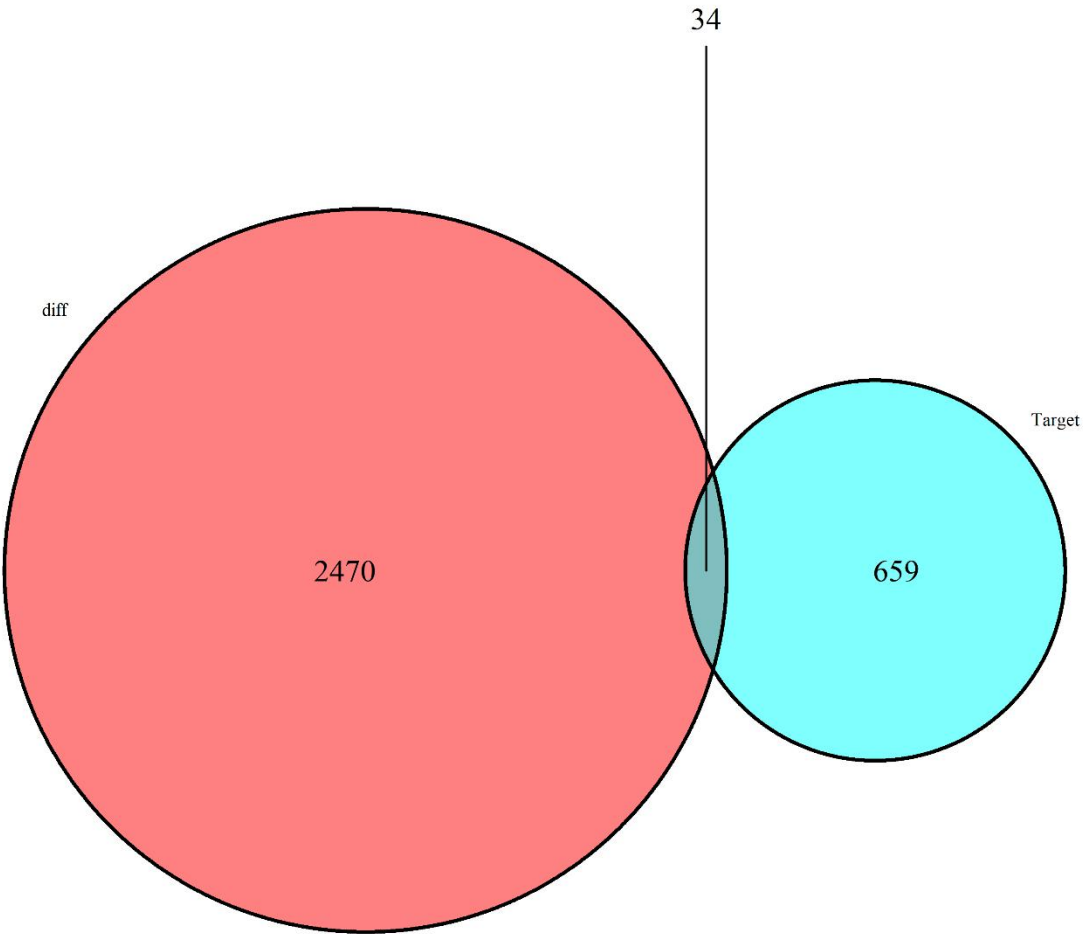

Supplement: Supplementary 1 — Supplemental Figure S1: Venn diagrams of differentially expressed microRNA targets. When matched to differentially expressed genes, 34 target genes were screened out. [file 4522210.f1.pdf]

Supplemental Figure S2

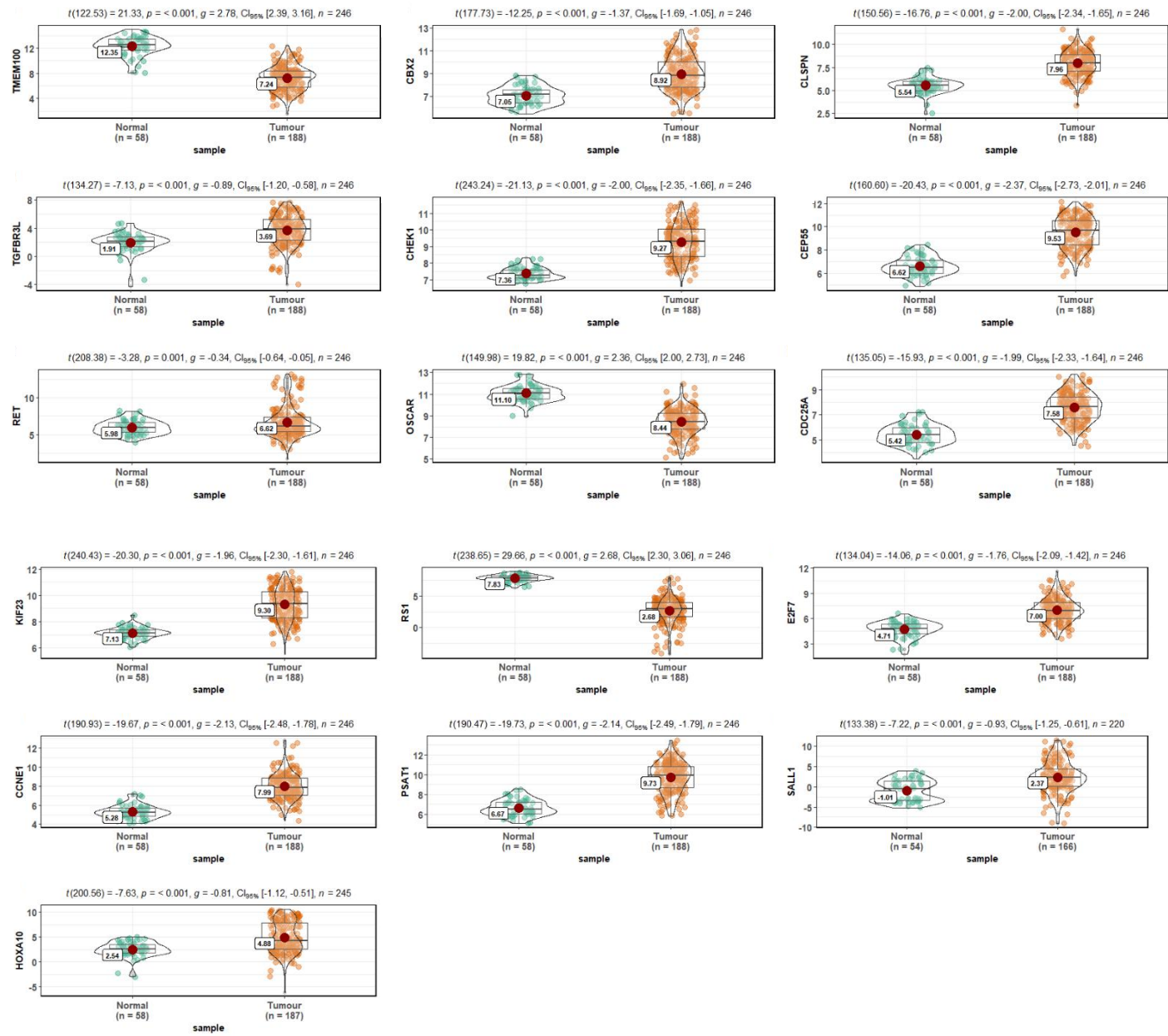

Supplement: Supplementary 2 — Supplemental Figure S2: expression of the target genes was plotted for LUAD tumor and normal tissues (TCGA dataset). Except TMEM00, RS1, and OSCAR, the remaining genes (CEP55, PSAT1, CHEK1, KIF23, CCNE1, CLSPN, CDC25A, E2F7, CBX2, HOXA10, SALL1, TGFBR3, and RET) were upregulated in LUAD tissues. [file 4522210.f2.pdf]

Supplemental Figure S3

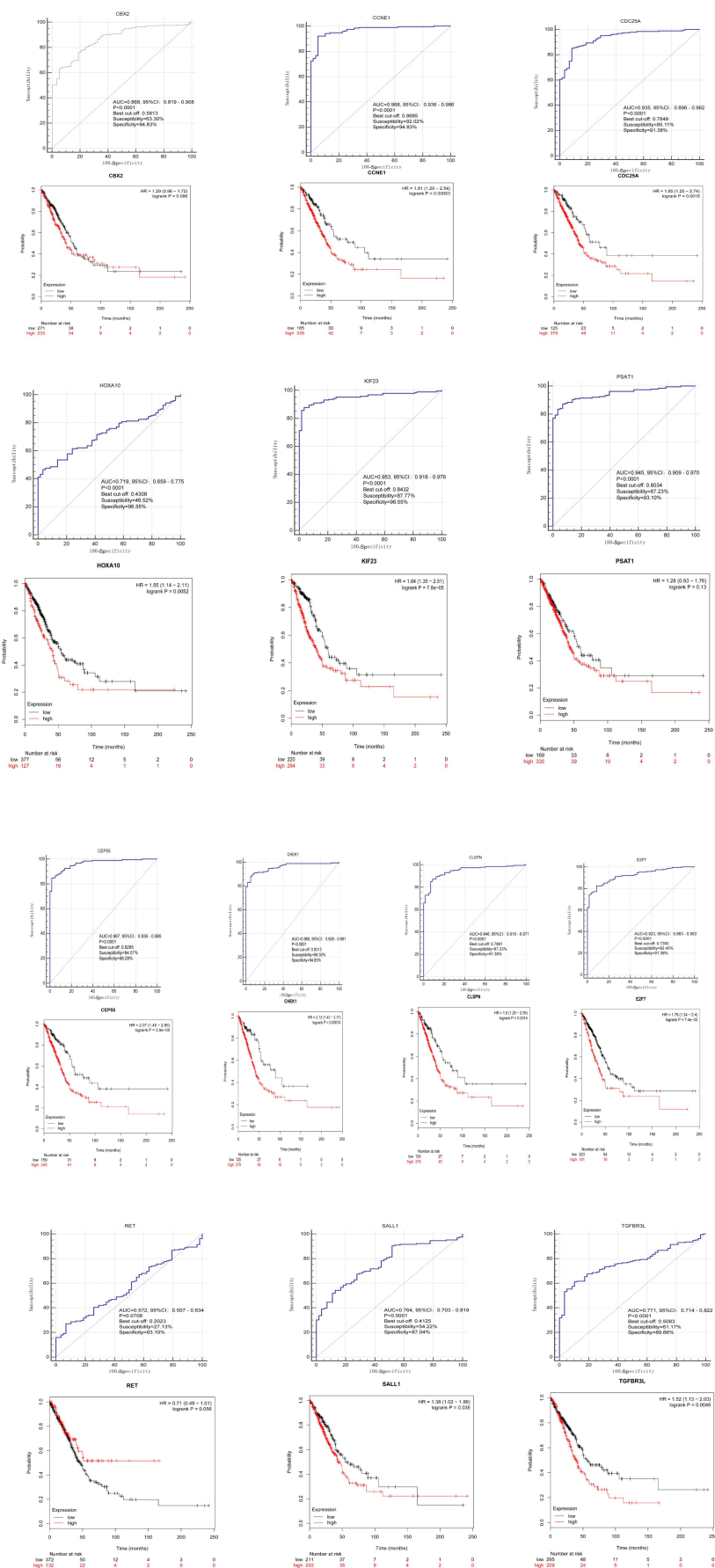

Supplement: Supplementary 3 — Supplemental Figure S3: the target genes were evaluated by diagnostic efficacy and prognostic analysis. ROC curve analysis was performed based on TCGA dataset. Kaplan-Meier survival curves of the overall survival were shown in the Kaplan-Meier plotter (https://kmplot.com/analysis/). [file 4522210.f3.pdf]
